# Reply to Mr. Jones on Medical Education

**Published:** 1813-06

**Authors:** 


*To the Editors of the Medical and Physical Journal.*

GENTLEMEN,

EVERY "unprejudiced mind" is, doubtless, a mind that thinks with Mr. Jones. As, however, from the purest of all religions there are heretics, and as men will most absurdly persist in thinking for themselves, he must permit me, "with all my selfish feelings about me," to examine some parts of his letter, which, from having appeared in your Journal, certainly deserves attention.

Mr. Jones very properly laments that there should be any disputes in a liberal profession, or that they should be attended by a want of liberality; then forthwith plunges into the thickest of the controversy he has just been deprecating, and scatters his abuse, with no unsparing hand, upon all who have the misfortune of differing from himself. To answer this abuse, would be to show myself as weak as the person who employs it. I leave him to his suspicions, and to his "imaginary opposition." Common-place sentiments of liberality, and "freedom from prejudice," are to be found in every school-boy's theme; they are the very pop-guns of controversy; a noise is made, but no impression is produced.

But it seems that to expose the deficiencies of the surgeon-apothecaries, is to diminish the character of the physician, because "we are membra ejusdem corporis." Poor Partridge himself never hit upon a happier non sequitur. "Sir," says the cock-crower to Mr. Young in Hamlet, "you must not complain of my not having crowed well: we are both performers, and by so doing you will lessen your reputation in that character." Mr. Jones details with much simplicity the duties of each department of the profession. How great a loss to his cause that having begun so well, he should so suddenly stop! It was to prevent the interference of these departments, that I have employed my pen against the Apothecaries' Bill. As a body, I consider the medical profession could not exist without them. For many of the individuals I entertain the highest respect. There are among them, and these not few, men eminent by their industry, their talents, and their integrity; but this is not the question, and since Mr. Jones has not thought proper to touch upon it, and this is merely intended as a letter of reply, I shall await a more favorable opportunity of expressing my further sentiments.

Admitting my ignorance of the requisites for constituting a respectable "surgeon-apothecary," I confess myself not much enlightened by the information that this gentleman from Guisbro', in the plenitude of his knowledge, has communicated; nor do I exactly see how a man can, or "ought to be forced by law to receive advantages," or to be "made respectable." His account of the education of these young gentlemen is too loose to be of any avail; but does he really imagine that the three winters and the summer at the university is spent in fiddling or jumping over the benches of the lecture-room? Far different are the pursuits of the alumni: they are obliged to be "Nec studio Citharæ, nec Musæ deditus ulli." Should he ever venture, for the purpose of graduation, into these regions of learning, he will find "more things than are dreamt of in his philosophy;" and still that this is not "all" required by the College of Physicians. I had intended to have entered upon an analysis of the *two educations*, (for, notwithstanding my Yorkshire friend's surmises, I am well acquainted with both,) but I fear to trespass upon your pages, and it is useless to point out to one who seems so well satisfied with the education that he doubtless has received, the superiority of any other. For a proof that the best is not without faults, I refer the incredulous to the letter of Mr. Jones.

I owe

I owe an apology to your readers and yourselves for delaying the consideration of this great question, by merely replying to an individual. It is sometimes necessary, however, for ulterior proceedings, to clear the ground and repel the charge of illiberality. Perhaps I ought to have recollected that the appearance of my letter in your Journal, would be a sufficient vindication of the purity of my motives. I am, Gentlemen,

Your most obedient Servant,

SALUS PUBLICA.

